# Supplementary material for: A Minimal Physiologically Based Pharmacokinetic Model to Characterize CNS Distribution of Metronidazole in Neuro Care ICU Patients
Source: Antibiotics (Basel). 2022 Sep 22;11(10):1293. doi: 10.3390/antibiotics11101293 (PMC9598284; doi:10.3390/antibiotics11101293)
Supplement: Supplementary file 1 [file antibiotics-11-01293-s001.zip › antibiotics-1836044-supplementary.pdf]

antibiotics

Supplementary

A Minimal Physiologically Based Pharmacokinetic Model to Characterize CNS Distribution of Metronidazole in Neuro Care ICU Patients

Alexia Chauzy [1], Salim Bouchène [2], Vincent Aranzana-Climent [1], Jonathan Clarhaut [1,3], Christophe Adier [1,3], Nicolas Grégoire [1,3], William Couet [1,3], Claire Dahyot-Fizelier [1,4] and Sandrine Marchand [1,3,*]

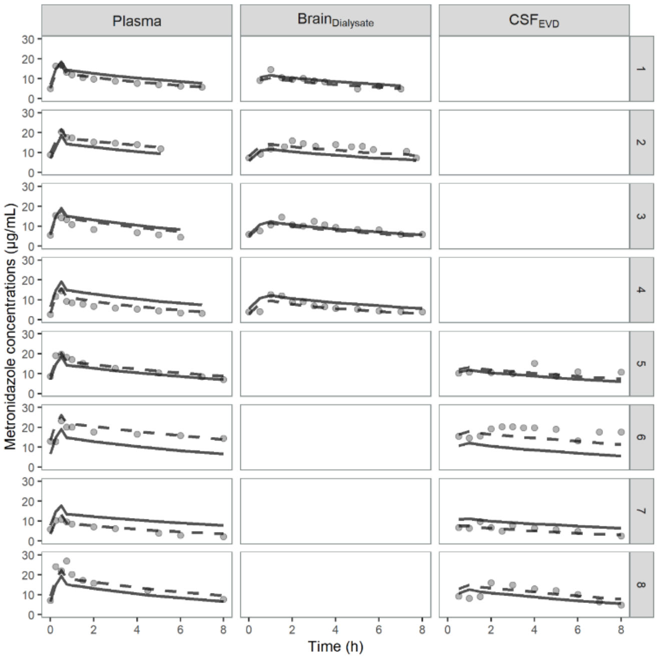

Figure S1. Metronidazole concentration-time profiles in plasma, brain dialysates after correction by *in vivo* probe recovery (Brain$_{Dialysate}$) and collection bag of the EVD (CSF$_{EVD}$) after the administration of 500 mg q8h. The circles represent the observed data, the dashed lines the individual predictions and the solid lines the population predictions.

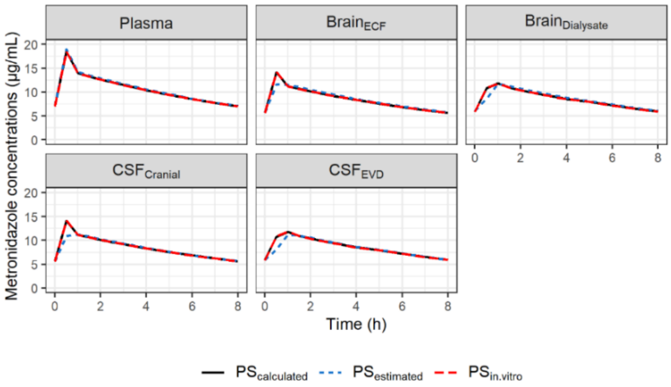

Figure S2. Predicted concentration-time profiles of metronidazole in plasma, brain ECF, brain dialysates after correction by *in vivo* probe recovery, CSF in the lateral ventricle (CSF$_{LV}$) and CSF collected by extra-ventricular drainage (CSF$_{EVD}$), for a typical patient after administration of 500 mg q8h. The black lines correspond to model predictions from permeability values (PS$_{ECF/CSF}$) calculated from system- and drug-specific parameters by Simcyp (PS$_{calculated}$), the blue dashed lines are model

predictions from $PS_{ECF/CSF}$ estimated by the model ($PS_{estimated}$) and the red dashed lines are model predictions from $PS_{ECF/CSF}$ scaled from *in vitro* Caco-2 permeability parameters ($PS_{in\ vitro}$).

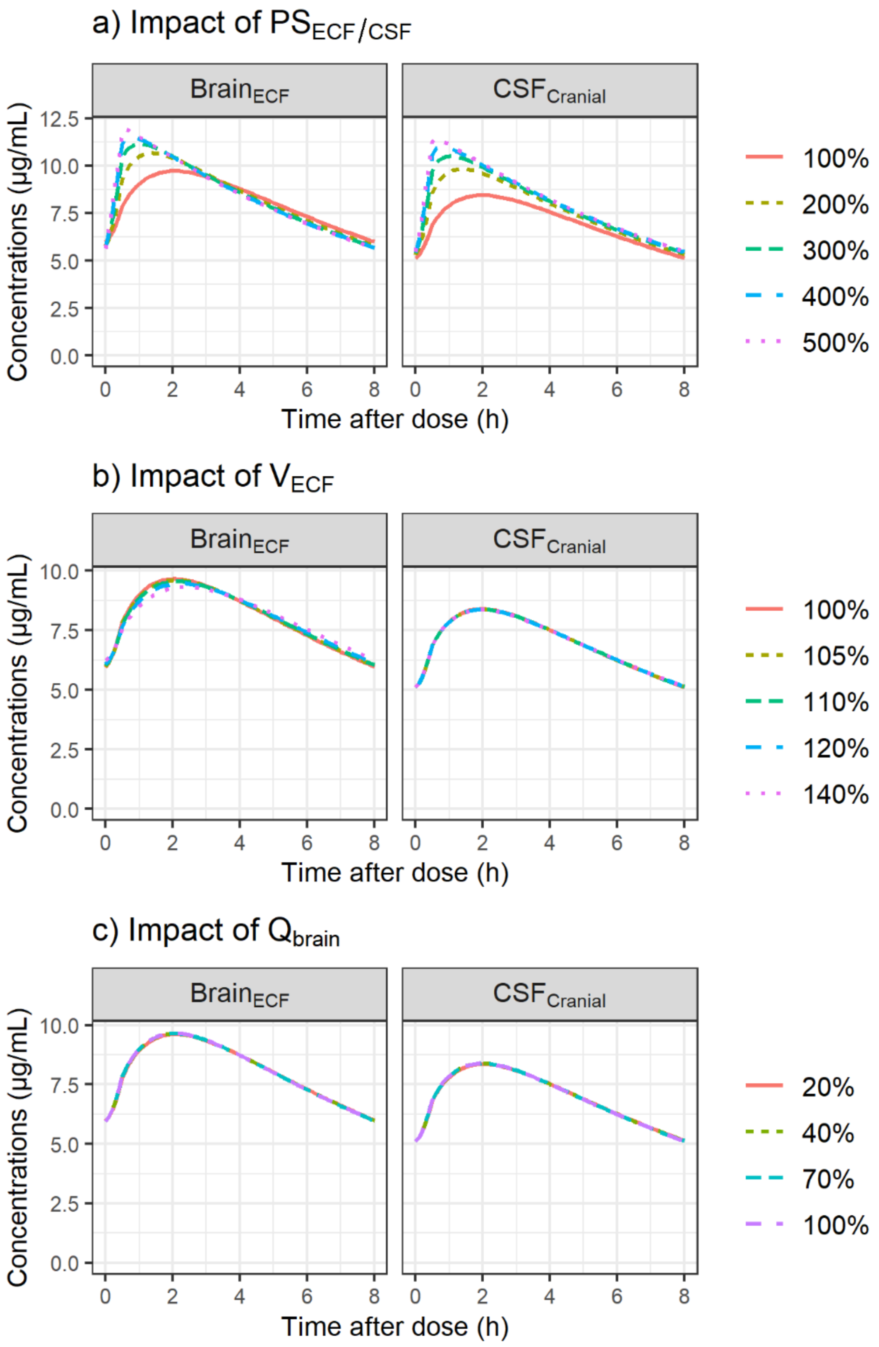

Figure S3. Simulation of the impact of increased passive diffusion clearances, $PS_{ECF/CSF}$ (a), increased brain ECF volume, V_{ECF} (b) and reduced cerebral blood flow, Q_{brain} (c) on CNS PK profiles of an hypothetical drug with low permeability using the minimal PBPK model. The plots were stratified by the CNS compartments (panels).

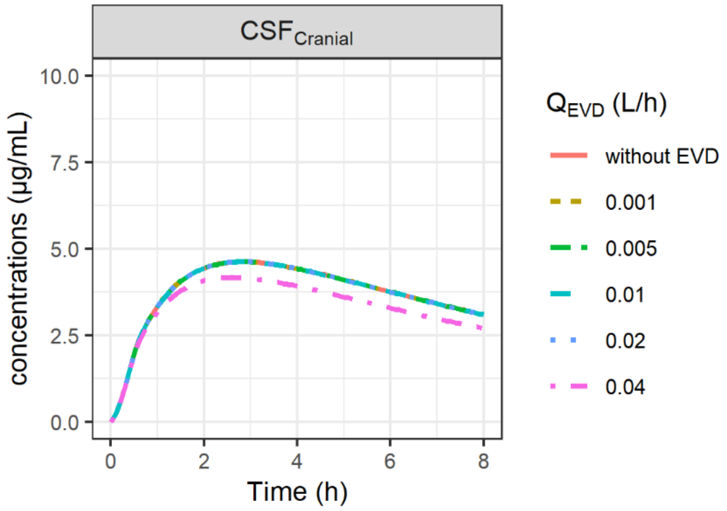

Figure S4. Simulation of the impact of external ventricular drain flow (Q_{EVD}) on CSF concentrations of an hypothetical drug with low permeability. Q_{EVD} was assumed to be constant over time and to restore the physiological CSF outflow (Q_{sink}). Thus when Q_{EVD} was higher than the physiological value of Q_{sink} (Q_{EVD} > 0.024 L/h), Q_{sink} was set at 0 L/h.

Table S1. Comparison between predicted and observed unbound AUCs between two consecutive dosing at steady-state (AUCΔt) in plasma, brain dialysate (BrainDialysate) and CSF collected by external ventricular drainage (CSFEVD).

	$AUC_{\Delta t}$ (µg/mL.h)					
	Plasma		$Brain_{Dialysate}$		CSF_{EVD}	
Patient	Observed	Predicted	Observed	Predicted	Observed	Predicted
1	68.0	70.6	59.5	56.3	-	56.0
2	113.7	111.4	94.8	88.9	-	88.4
3	57.4	78.0	71.0	62.3	-	61.9
4	45.2	57.2	50.5	45.7	-	45.4
5	96.0	100.5	-	80.2	87.6	79.8
6	134.2	147.3	-	117.6	140.4	116.9
7	52.9	50.0	-	40.0	42.1	39.7
8	122.3	109.7	-	87.6	82.1	87.1
mean	86.2	90.6	68.9	72.3	88.1	71.9
mean fold error		1.05		1.05		0.82

Table S2. Comparison of parameter values when PS values are calculated from system- and drug-specific paramaters ($PS_{calculated}$) using Simcyp or estimated by the model ($PS_{estimated}$).

	$PS_{calculated}$		$PS_{estimated}$	
**Parameter**	**Value (95% CI)** [a]	**IIV %CV (95% CI)** [a]	**Value (95% CI)** [a]	**IIV %CV (95% CI)** [a]
K_p	0.796 (0.693-0.923)	-	0.767 (0.671-0.888)	-
CL_{10} (L/h)	7.28 (5.77-9.53)	35.2 (24.0-57.8)	7.18 (5.60-9.16)	34.8 (24.2-52.9)
**fd**	0.86 FIX[b]		0.823 (0.616-0.976)	-
PS_{ECF} **(L/h)**	6.4 FIX[c]	-	0.904 (0.560-1.87)	-
PS_{CSF} **(L/h)**	3.2 FIX[d]	-	0.398 (0.175-1.06)	-
$\sigma_{prop,plasma}$ **(%)**	14.4 (9.74-19.1)	-	14.8 (11.0-19.3)	-
$\sigma_{add,plasma}$ **(μg/mL)**	1.18 (0.320-2.90)	-	1.25 (0.364-2.57)	-
$\sigma_{prop,ECF}$ **(%)**	22.8 (17.5-29.1)	-	19.4 (14.8-26.4)	-
$\sigma_{prop,CSF}$ **(%)**	28.2 (22.0-36.5)	-	27.4 (21.2-35.9)	-

IIV: Inter-individual Variability; CV: Coefficient of Variation; CI: Confidence Interval; [a] The 95% CI was obtained by Sampling Importance Resampling. [b] Parameter fixed to the maximum allowed value due to identifiability issue; [c] Parameter fixed to the value predicted by Simcyp based on system (surface area of BBB) and drug-specific parameters (log P and molecular weight); [d] PS_{CSF} was assumed to be half of PS_{ECF}.

Table S3. EVD experimental data.

**Sampling time**	**Time interval (h)**	V_{EVD} **(mL)**	Q_{EVD} **(L/h)**	C_{EVD} **(µg/mL)**	A_{EVD} **(µg)**
**Patient 1**					
0-0.5h	0.5	1.5	0.003	10.1	15.2
0.5-1h	0.5	0.5	0.001	10.7	5.36
1-1.5h	0.5	0	0	0	0
1.5-2h	0.5	1.5	0.003	10.6	15.8
2-3h	1	16	0.016	10.1	161.3
3-4h	1	2.5	0.0025	15.2	38.1
4-5h	1	9	0.009	8.81	79.3
5-6h	1	13	0.013	11.0	143
6-7h	1	nd	nd	nd	nd
7-8h	1	19	0.019	10.7	202
**Total**					**660**
**Patient 2**					
**Sampling time**	**Time interval (h)**	V_{EVD} **(mL)**	Q_{EVD} **(L/h)**	C_{EVD} **(µg/mL)**	A_{EVD} **(µg)**
0-0.5h	0.5	2.5	0.005	15.5	38.8
0.5-1h	0.5	3.5	0.007	14.7	51.4
1-1.5h	0.5	3	0.006	15.8	47.3
1.5-2h	0.5	20	0.040	19.2	385
2-2.5h	0.5	17	0.034	20.4	346
2.5-3h	0.5	9	0.018	20.3	183
3-3.5h	0.5	6	0.012	19.7	118
3.5-4h	0.5	6	0.012	19.9	119
4-5h	1	11	0.011	19.2	211
5-6h	1	10	0.010	13.2	132
6-7h	1	7	0.007	17.7	124
7-8h	1	4.5	0.0045	17.7	79.8
**Total**					**1835**
**Patient 3**					
**Sampling time**	**Time interval (h)**	V_{EVD} **(mL)**	Q_{EVD} **(L/h)**	C_{EVD} **(µg/mL)**	A_{EVD} **(µg)**
0-0.5h	0.5	4	0.008	6.85	27.4
0.5-1h	0.5	4	0.008	6.28	25.1
1-1.5h	0.5	5	0.010	9.66	48.3
1.5-2h	0.5	8.5	0.017	6.68	56.8
2-2.5h	0.5	4.5	0.009	4.99	22.4
2.5-3h	0.5	4	0.008	7.80	31.2
3-3.5h	0.5	na	Na	na	na
3.5-4h	0.5	7	0.014	6.42	44.9
4-5h	1	2	0.002	5.59	11.2
5-6h	1	13	0.013	4.70	61.1
6-7h	1	3	0.003	.	na
7-8h	1	7	0.007	2.43	17.0
**Total**					**345**
**Patient 4**					
**Sampling time**	**Time interval (h)**	V_{EVD} **(mL)**	Q_{EVD} **(L/h)**	C_{EVD} **(µg/mL)**	A_{EVD} **(µg)**
0-0.5h	0.5	6.5	0.013	9.23	60.0

0.5-1h	0.5	5	0.010	8.14	40.7
1-1.5h	0.5	5	0.010	9.03	45.2
1.5-2h	0.5	3	0.006	16.0	48.1
2-3h	1	12	0.012	14.9	178.9
3-4h	1	15	0.015	12.9	193.7
4-5h	1	10	0.010	12.1	121.2
5-6h	1	15	0.015	10.1	151.9
6-7h	1	10	0.010	6.50	65.0
7-8h	1	6	0.006	4.91	29.4
**Total**					**934**
